# PN-ImTLSM facilitates high-throughput low background single-molecule localization microscopy deep in the cell

**DOI:** 10.52601/bpr.2021.210014

**Published:** 2021-08-31

**Authors:** Boxin Xue, Caiwei Zhou, Yizhi Qin, Yongzheng Li, Yuao Sun, Lei Chang, Shipeng Shao, Yongliang Li, Mengling Zhang, Chaoying Sun, Renxi He, Qian Peter Su, Yujie Sun

**Affiliations:** 1 State Key Laboratory of Membrane Biology, Biomedical Pioneer Innovation Center (BIOPIC), School of Life Sciences, Peking University, Beijing 100871, China; 2 Academy for Advanced Interdisciplinary Studies, Peking University, Beijing 100871, China; 3 School of Future Technology, Peking University, Beijing 100871, China; 4 College of Chemistry and Molecular Engineering, Peking University, Beijing 100871, China; 5 Bioland Laboratory (Guangzhou Regenerative Medicine and Health Guangdong Laboratory), Guangzhou 510005, China; 6 Beijing Institute of Heart Lung and Blood Vessel Disease, Beijing Anzhen Hospital, Capital Medical University, Beijing 100029, China; 7 Department of Geriatric Dentistry, Peking University School and Hospital of Stomatology, Beijing 100081, China; 8 School of Biomedical Engineering, Faculty of Engineering and Information Technology, University of Technology Sydney, Sydney, NSW 2007, Australia

**Keywords:** Light-sheet fluorescence microscopy, Background-free fluorescence imaging, Deep learning, Single cell imaging, Homogeneous illumination, Single-molecule localization microscopy

## Abstract

When imaging the nucleus structure of a cell, the out-of-focus fluorescence acts as background and hinders the detection of weak signals. Light-sheet fluorescence microscopy (LSFM) is a wide-field imaging approach which has the best of both background removal and imaging speed. However, the commonly adopted orthogonal excitation/detection scheme is hard to be applied to single-cell imaging due to steric hindrance. For LSFMs capable of high spatiotemporal single-cell imaging, the complex instrument design and operation largely limit their throughput of data collection. Here, we propose an approach for high-throughput background-free fluorescence imaging of single cells facilitated by the Immersion Tilted Light Sheet Microscopy (ImTLSM). ImTLSM is based on a light-sheet projected off the optical axis of a water immersion objective. With the illumination objective and the detection objective placed opposingly, ImTLSM can rapidly patrol and optically section multiple individual cells while maintaining single-molecule detection sensitivity and resolution. Further, the simplicity and robustness of ImTLSM in operation and maintenance enables high-throughput image collection to establish background removal datasets for deep learning. Using a deep learning model to train the mapping from epi-illumination images to ImTLSM illumination images, namely PN-ImTLSM, we demonstrated cross-modality fluorescence imaging, transforming the epi-illumination image to approach the background removal performance obtained with ImTLSM. We demonstrated that PN-ImTLSM can be generalized to large-field homogeneous illumination imaging, thereby further improving the imaging throughput. In addition, compared to commonly used background removal methods, PN-ImTLSM showed much better performance for areas where the background intensity changes sharply in space, facilitating high-density single-molecule localization microscopy. In summary, PN-ImTLSM paves the way for background-free fluorescence imaging on ordinary inverted microscopes.

## INTRODUCTION

Out-of-focus fluorescence is difficult to model due to its heterogeneity, complexity, and lack of prior information. Total internal reflection fluorescence microscopy (TIRFM) uses the exponential attenuation propagation characteristics of evanescent waves along *z*-axis to reduce out-of-focus background (Axelrod 1981). However, when imaging the cell nucleus, low signal-to-background ratio (SBR) becomes an important obstacle because the imaging depth exceeds the inherent 200 nm range of TIRFM. Light-sheet illumination is a wide-field imaging method with fast imaging speed and low out-of-focus background (Shao *et al*. 2018). The orthogonal excitation and detection scheme can produce unprecedented image contrast (Huisken *et al*. 2004; Keller *et al*. 2008; Siedentopf and Zsigmondy 1902), making it widely used in tissue imaging. However, geometric obstacles hinder the combination of thin light sheets (full width at half maximum (FWHM) approximately 1 micron) and high numerical aperture (NA) detection objectives (above 1.40), resulting in a trade-off between high SBR and high fluorescence collection efficiency, both of which are required for intra-nucleus imaging.

Various light sheet methods have been developed to deal with geometric obstacles (Bouchard *et al*. 2015; Chen *et al*. 2014; Galland *et al*. 2015; Gebhardt *et al*. 2013; Gustavsson *et al*. 2018; Manton and Rees 2016; Strnad *et al*. 2016; Theer *et al*. 2016). Reflected light-sheet microscopy (RLSM) uses an AFM cantilever as a micromirror to reflect the light sheet, so that the high NA illumination objective and the detection objective are opposingly aligned (Gebhardt *et al*. 2013). Lattice light-sheet microscopy (LLSM) uses a custom-made immersion objective pair to break the geometric barriers, but at the expense of detection NA (1.1) (Chen *et al*. 2014). Tilted light sheet microscopy with 3D point spread functions (TILT3D) uses a long working distance air objective and a prism set to guide the light-sheet into the sample (Gustavsson *et al*. 2018). The relatively thick light-sheet is matched by the detection depth expanded using PSF engineering technology. Although the aforementioned techniques have shown excellent performance for single cell imaging, the complexity of instrument operation and maintenance greatly limits their throughput for massive data collection. One attempt to overcome this obstacle is through single-objective based systems (Kim *et al*. 2019; Yang *et al*. 2019), which are compatible with multi-well plates. However, loss of fluorescence due to additional optical components is a problem, especially when dealing with weak signals. The inclined nature also limits the combination with large field of view (FOV) excitation under high NA collection. In short, the wide application of these existing methods is limited by their complexity and low-throughput nature.

The rise of data-driven methods such as deep learning provides us with a new perspective to solve this problem (Wang *et al*. 2019; Barbastathis *et al*. 2019; Belthangady and Royer 2019). We emphasize that background removal based on light-sheet illumination can be regarded as a ground truth for training deep learning networks. In fact, deep learning has been combined with light-sheet illumination to successfully handle tissue imaging (Bai *et al*. 2019; Corsetti *et al*. 2020; Fang *et al*. 2021; Geng *et al*. 2021; Zhao *et al*. 2020). However, deep learning models for single cell background-free fluorescence imaging were only trained on simulated datasets (Möckl *et al*. 2020) due to lack of a light-sheet imaging scheme that simultaneously has high-throughput data collection and high imaging sensitivity.

Here, we introduce the immersion tilted light sheet microscopy (ImTLSM), which has high SBR and high fluorescence collection efficiency. ImTLSM greatly reduces the difficulties in use and maintenance, so that high-throughput image collection can be performed. This combination allows us to collect a large number of high-quality deep learning datasets. Thereby, we establish a background removal dataset based on epi-illumination and ImTLSM illumination, and train a deep learning model to map epi-illumination images to ImTLSM illumination images, which paves the way for background-free fluorescence imaging on ordinary inverted microscopes. We name this method PN-ImTLSM and show that it can be generalized to large-field homogeneous illumination imaging to further enhance the throughput. When applied to single-molecule localization microscopy (SMLM), we found that the background removal effect of PN-ImTLSM was better than ordinary methods, especially for areas where the background intensity changed sharply in space. The acquisition speed of high-precision points has been doubled, which further improves the throughput of accurate data collection.

## RESULTS AND DISCUSSION

### Characterization of ImTLSM performance

**Figure 1:**
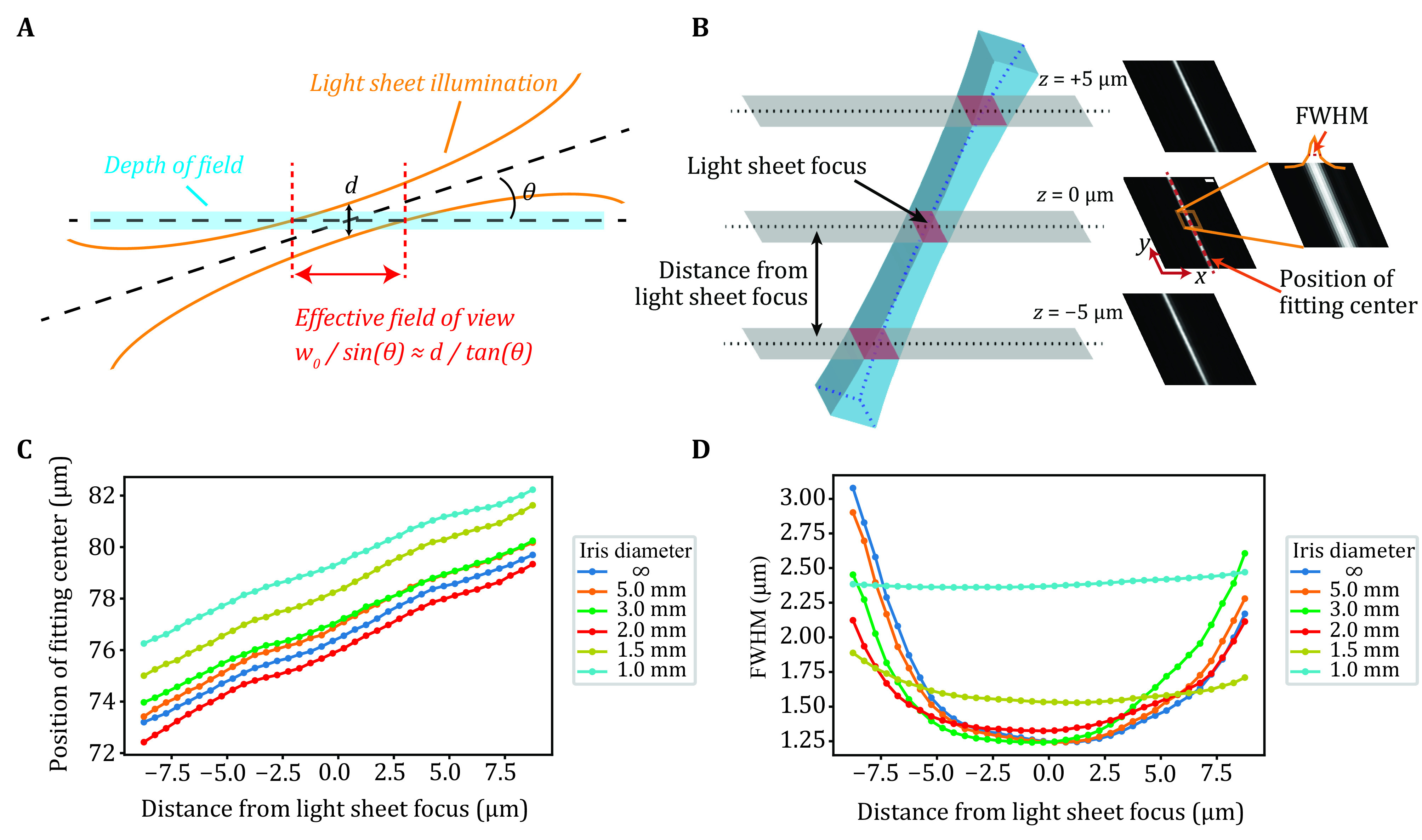
Calibration of ImTLSM illumination profile. **A** Schematic diagram showing the illumination geometry of ImTLSM. The effective field of view (eFOV) is defined as the range inside which the center focal plane is effectively illuminated by the light sheet (inside the beam waist of light sheet). **B** Calibration method. To calibrate the light sheet profile, we changed the offset direction of illumination objective such that the light sheet tilted angle was the surplus of *θ*. Light sheet profile images were taken using EMCCD directly under weak 560 nm laser illumination, with detection objective scan along *z*-axis (500 nm step size). FWHM and the position of fitting center were calculated from these images. **C** Positions of fitting centers as a function of the distance from light sheet focus. The estimated focal plane under different iris open conditions was plotted as the zero point. The slopes of fitting curves for each iris diameter were nearly identical, showing approximately 18 degrees of inclination in each case. **D** FWHM of light sheet as a function of the distance from light sheet focus

The illumination geometry from ImTLSM is shown in Fig. 1A. Since the light-sheet illumination is tilted, the region in the focal plane where the molecules could be effectively excited by the light sheet would be determined by the thickness (*i.e*., the beam waist *w*_0_) of the light sheet and the tilted angle *θ* of the light sheet. We thereby defined this region as the effective field of view (eFOV). To measure *w*_0_ and *θ*, the light sheet was projected directly to an EMCCD and the intensity profile of the illumination was obtained by measuring the FWHMs of the illumination cross-section at different distances relative to the focal plane (Fig. 1B and supplementary Fig. S1). As *w*_0_ and *θ* may both be dependent on the iris opening size, we measured the illumination intensity profiles under different iris opening sizes. As shown in Fig. 1C, the tilted angle was about 18 degrees under all iris sizes. In contrast, *w*_0_ was found to be highly dependent on the iris sizes (Fig. 1D). When the iris was left open for full ability of background reduction, the light sheet became the thinnest (FWHM ≈ 1.25 μm), albeit with a limited field of view (eFOV ≈ 4 μm). When the iris was closed to a diameter of 1.0 mm, its eFOV became the largest (eFOV ≈ 8 μm) with a FWHM of about 2.4 μm. Importantly, when the iris was closed to smaller than 2.0 mm in diameter, the intensity profile did not change significantly as the focusing plane slightly moved up and down (Fig. 1B and 1D, supplementary Fig. S1), enabling a robust illumination condition for finding the focal plane when switching from one sample to another for data collection of a large amount of cells. In practice, we found that 1.5 mm iris diameter mode was the best in balance of light sheet thickness (FWHM ≈ 1.6 μm) and eFOV (~5 μm).

**Figure 2:**
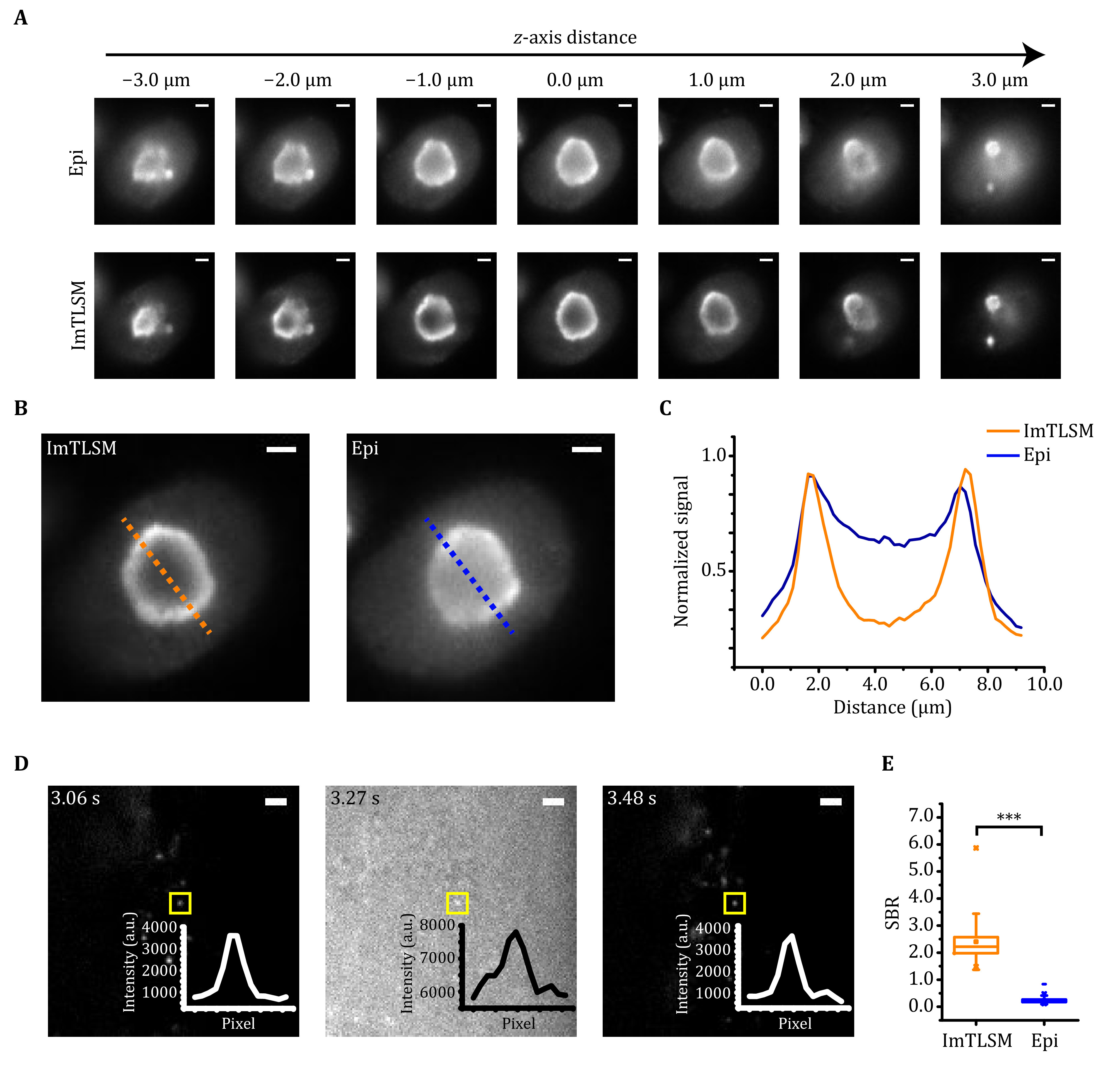
Background removal performance of ImTLSM. **A** Comparison between epi-illumination (Epi) and light-sheet illumination (ImTLSM) at different *z*-axis positions throughout a cell nucleolus. **B** Immuno-fluorescence labelled NCL protein distribution. Images were taken under different illumination modes (left: ImTLSM (iris open), right: epi-illumination, scale bar: 2 μm). **C** Intensity profile from dashed lines on **B** (orange solid line: ImTLSM, blue solid line: epi-illumination). **D** Alternatingly collected ImTLSM and epi-illumination images of a HeLa cell expressing YAP–HaloTag with 30 ms time resolution (iris open. Scale bar: 2 μm). **E** SBR statistics under different imaging modes from **D** (*** stands for *p*-value < 0.001)

We next evaluated the performance of ImTLSM in terms of its background reduction capability and data collection efficiency for imaging multiple cells. The background reduction power of ImTLSM was evaluated by measuring the SBR of immuno-fluorescently labelled NCL protein, which are distributed at the surface of the nucleolus (Fig. 2A–2C). As nucleoli are located deep in nuclei, epi-illumination of NCL led to high fluorescence background (Fig. 2A). In contrast, ImTLSM was able to clearly resolve the hollow distribution of NCL (Fig. 2A). An enhancement in SBR was about 2.3 when comparing ImTLSM (FWHM ≈ 1.25 μm) to epi-illumination (Fig. 2B and 2C), with a median value of about 1.9 from 30 cells (supplementary Fig. S2). We also imaged Lamin B1 and Nup133 protein, which are distributed on the nuclear envelop. The results also showed clear enhancement in SBR for both samples imaged by ImTLSM versus epi-illumination (supplementary Fig. S3). We further tested the SBR enhancement for intra-nuclear single-molecule imaging, which is generally quite challenging due to extremely poor SBR under epi-illumination mode. We expressed YAP–HaloTag in HeLa cells and imaged the same individual molecules by alternating ImTLSM (iris fully opened) and epi-illumination. We found that SBR of single molecule imaged with epi-illumination was only 0.25, hardly sufficing to distinguish single molecules from the background noise. In striking contrast, the SBR of single molecule imaged with ImTLSM was about 2.30, leading to about 9-fold enhancement in SBR (Fig. 2D and 2E). These results indicated that ImTLSM is particularly suitable for single-molecule imaging in the nucleus.

To evaluate the data collection efficiency of ImTLSM for imaging multiple cells, we performed a fast scanning of Lamin B1-labeled cells (supplementary Movie S1). The high-throughput nature of ImTLSM enabled highly efficient imaging and data acquisition for deep learning training.

### Deep learning training performance using high-throughput imaging of ImTLSM

We took the advantage of the high-throughput imaging capability of ImTLSM to collect a large dataset for deep learning. To do so, we prepared immuno-stained H2B samples and collected fluorescence signal of tens of thousands single molecules over hundreds of cells. As H2B is densely distributed throughout the nucleus, the fluorescence background of immuno-stained H2B sample would be mainly from the out-of-focus fluorescence. Conversely, the fluorescence background of structural objects usually contains contribution from the morphological features besides the out-of-focus fluorescence, making the deep learning model less robust when being applied to background reduction of different structures.

**Figure 3:**
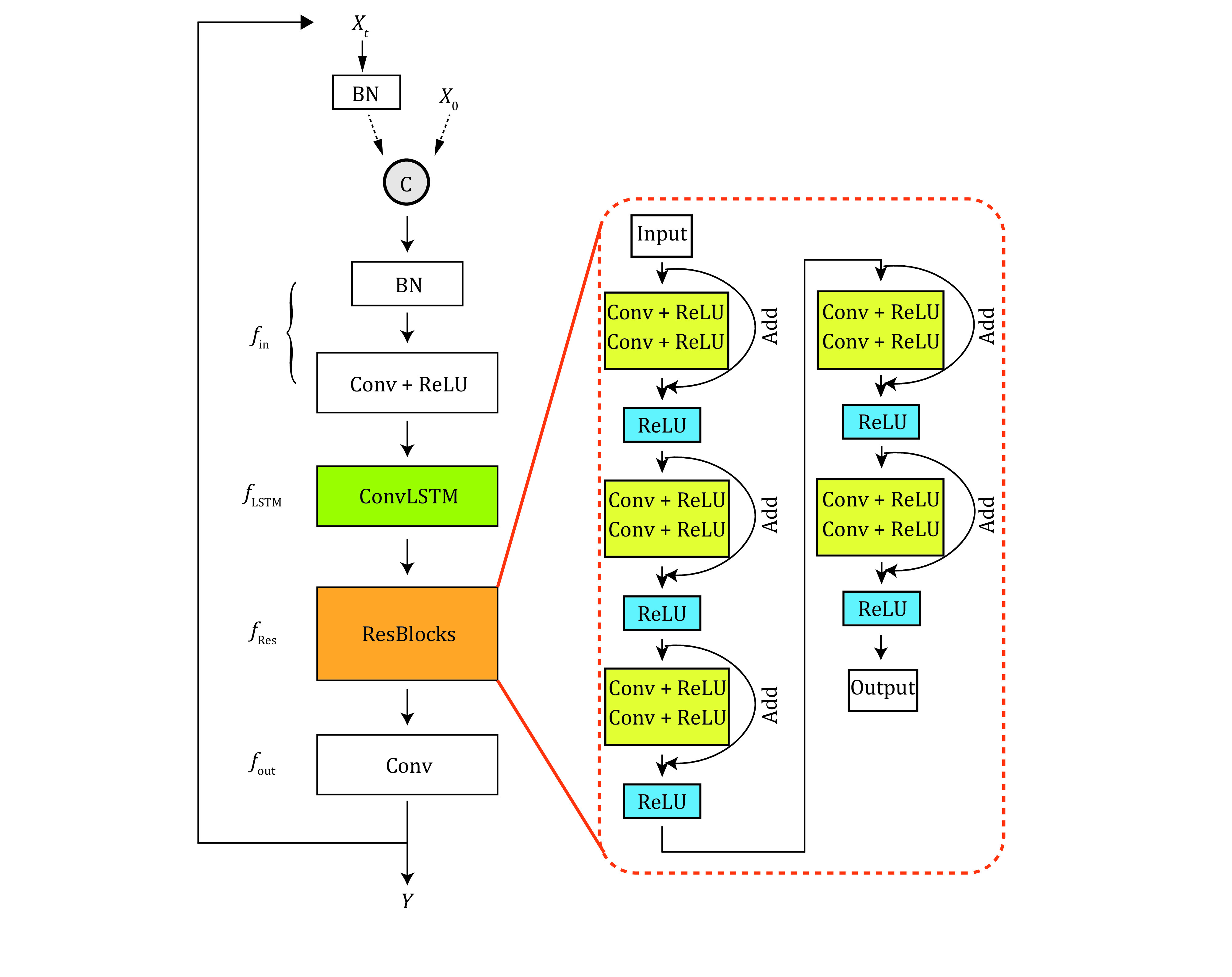
Modified PReNet structure. BN: batch normalization layer; Conv + ReLU: convolution layer with ReLU; ConvLSTM: convolutional LSTM; Add: addition operator

**Figure 4:**
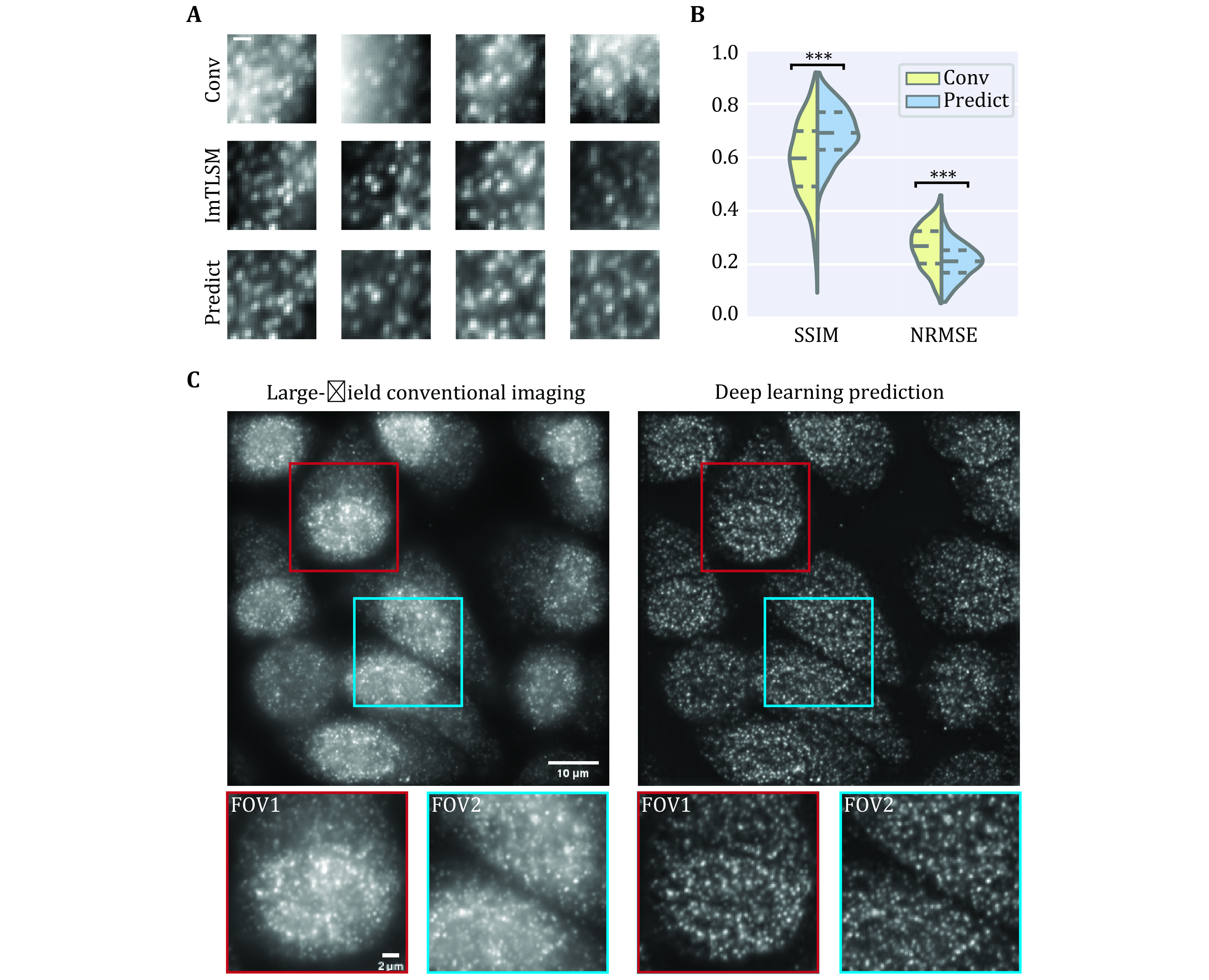
Deep learning prediction performance. **A** Comparison between epi-illumination results, ImTLSM results and deep learning prediction results (scale bar: 1 μm). **B** Structural similarity (SSIM) index and normalized root mean square error (NRMSE) statistics for Conv-ImTLSM image pairs and Predict-ImTLSM image pairs (***p < 0.001). **C** Performance of generalization to large field (left: large-field homogeneous illumination image, right: deep learning prediction result). Scale bar: 10 μm for large images and 2 μm for small images

As deep learning is sensitive to the noise level, we collected 100 frames for each FOV under epi-illumination mode and ImTLSM mode sequentially, which were then averaged as one pair of training datum to reduce the influence from Poisson-Gaussian noise. Using a modified PReNet to 2,400 such pairs of fluorescence images, we obtained an ImTLSM-facilitated background removal model named as PN-ImTLSM (Fig. 3). The results showed that the trained model was able to successfully reduce the background in the conventional images obtained by the epi-illumination mode and generate predicted images that were similar with that obtained by ImTLSM (Fig. 4A and 4B). While the molecule numbers detected under different modes were nearly the same, our quantification showed that both ImTLSM and deep learning images had about 2-fold enhanced SBR compared to the conventional images collected with epi-illumination.

Large FOVs are required for many biomedical fluorescence imaging applications. As ImTLSM has limited FOVs, we further tested the performance of the PN-ImTLSM model being generalized to large-field homogeneous illumination images. To achieve large field illumination, a large-field excitation system was custom built on the same microscope with ImTLSM (supplementary Fig. S4). As shown in Fig. 4C, the performance of large-field generalization was excellent, which greatly compensates the limited FOV of ImTLSM and extends the application of our method.

### Application on intra-nuclear super-resolution imaging

Next, we investigated whether our PN-ImTLSM model can be used to improve the performance of single-molecule localization microscopy (SMLM). To this end, we prepared a stable HeLa cell line expressing H2B-mMaple3, and collected SMLM images under the large-field homogeneous illumination scheme.

**Figure 5:**
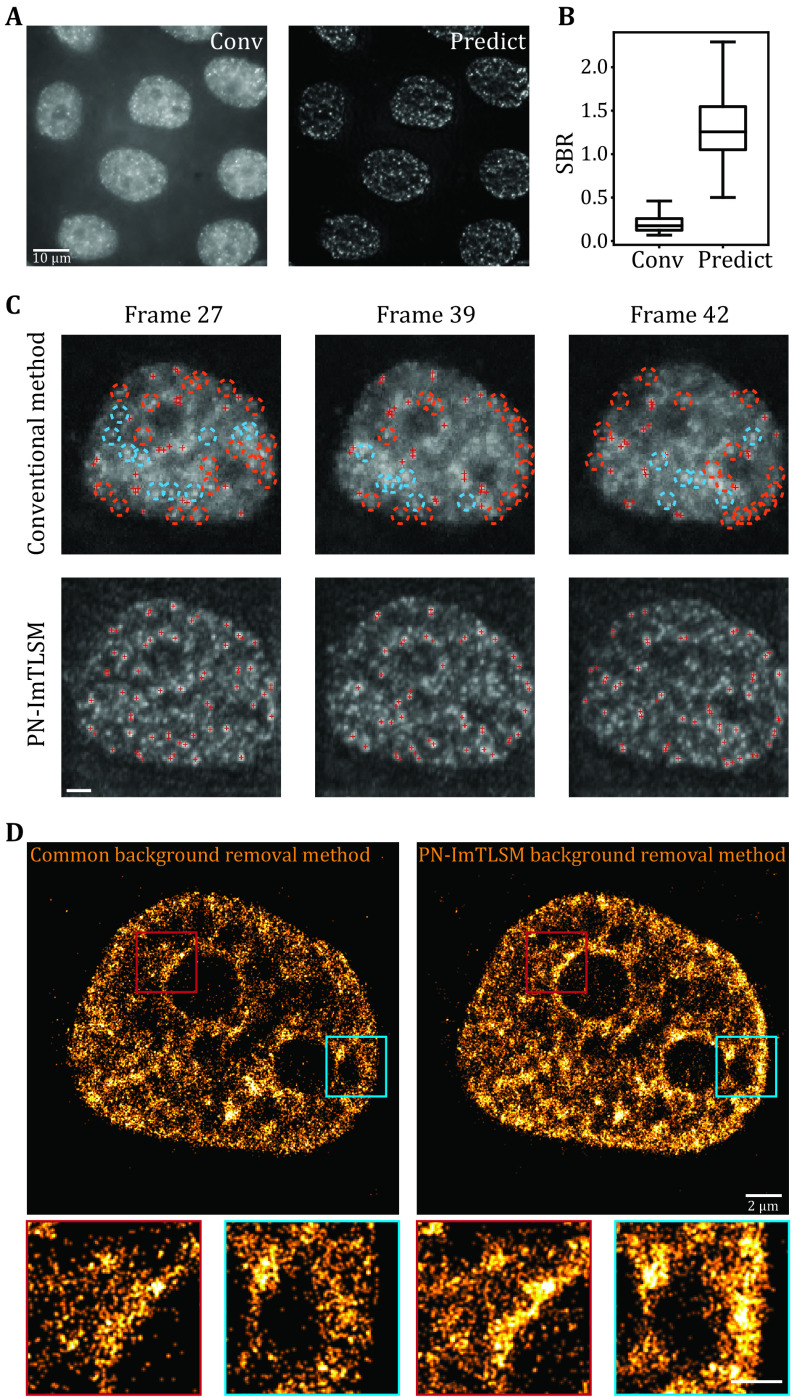
Performance enhancement in SMLM experiments. **A** Comparison between large-field homogeneous illumination SMLM image (left, scale bar: 10 μm) and deep learning predicted SMLM image (right). **B** SBR statistics for high localization precision fittings in **A**. **C** Performance comparison between conventional background removal method and PN-ImTLSM method in single molecule identification. Single-molecule localization was performed using ThunderSTORM with peak intensity threshold of 4*std (Wave.F1). Orange circles indicate missing localizations near the edge while blue circles indicate missing localizations in other positions. PN-ImTLSM demonstrates much better performance in areas where background changed rapidly, such as the edges of nucleus or nucleolus (scale bar: 2 μm). **D** Comparison between the conventional background removal method and PN-ImTLSM background removal method. The threshold in ThunderSTORM was set to 4*std to investigate the image reconstruction performance under high localization precision filtering. The enlarged image areas indicated that PN-ImTLSM background removal method performed well near edges where background level changed rapidly, while ImageJ plugin background removal method failed to remove such uneven background (scale bar: 2 μm for large images and 1 μm for small images)

We first used PURE-LET to denoise all frames to reduce Poisson-Gaussian noise (Li *et al*. 2018). Then, we applied the model to remove the background. As shown in Fig. 5, individual molecules that were difficult to identify in conventional epi-illumination images were reliably identified in the predicted images with enhanced SBR (Fig. 5A and 5B, supplementary Movie S2). To test the performance of our PN-ImTLSM model in SMLM super-resolution imaging, we applied the model to 10,000 raw image frames (Fig. 5D). In order to accurately reconstruct the structural details, we set the threshold in ThunderSTORM (Ovesný *et al*. 2014) to 4*std, which is 2 times higher than the generally recommended 2*std. Thus, we could compare the performance of retaining high localization precision points. Compared with the conventional method to subtract background in ImageJ, we found that using PN-ImTLSM to preserve localization is twice as effective. In other words, when processing high-density images, our method can retain twice as much accurate information in the same frame range, thereby increasing the acquisition speed of reconstructing the same structure by two-fold. In addition, we found that the number of localizations near the nucleus or nucleolus edge was dramatically enhanced. We studied each frame and found that the rapidly changing background level should be the main obstacle (Fig. 5C). To quantitate the enhancement of PN-ImTLSM in SMLM and the artifacts of the conventional method, we subtract the SMLM images obtained with the two methods (supplementary Fig. S5). PN-ImTLSM succeeded in reducing the uneven background even near the edges, while the ImageJ subtract background plugin method failed, leaving only a small amount of localizations (Fig. 5C and 5D, supplementary Fig. S5).

## DISCUSSION AND CONCLUSION

In summary, we have developed PN-ImTLSM, which can remove background for large FOV imaging under epi-illumination scheme. We applied PN-ImTLSM to high-density SMLM and found that for points with high localization precision, the acquisition speed was doubled. Compared with conventional background removal methods, our method performs particularly well when the background level changes rapidly, which is the case when imaging nucleus or nucleolus edge. PN-ImTLSM can also be used in combination with high-density algorithms to improve fitting accuracy. We found that the choice of denoising method is crucial for avoiding artifacts in different situations. Therefore, in practice, users should test different denoising methods for specific demands (supplementary Fig. S6). While we only demonstrated its application in SMLM experiments, we found that it has good generalization performance for fluorescence structure imaging (supplementary Fig. S7), further expanding its application. In addition, it is worth noting that because of its simple and robust configuration, ImTLSM can in principle be installed on any kind of inverted microscope, paving the way for background-free fluorescence imaging on ordinary microscopes.

## MATERIALS AND METHODS

### Immersion tilted light sheet microscopy (ImTLSM)

**Figure 6:**
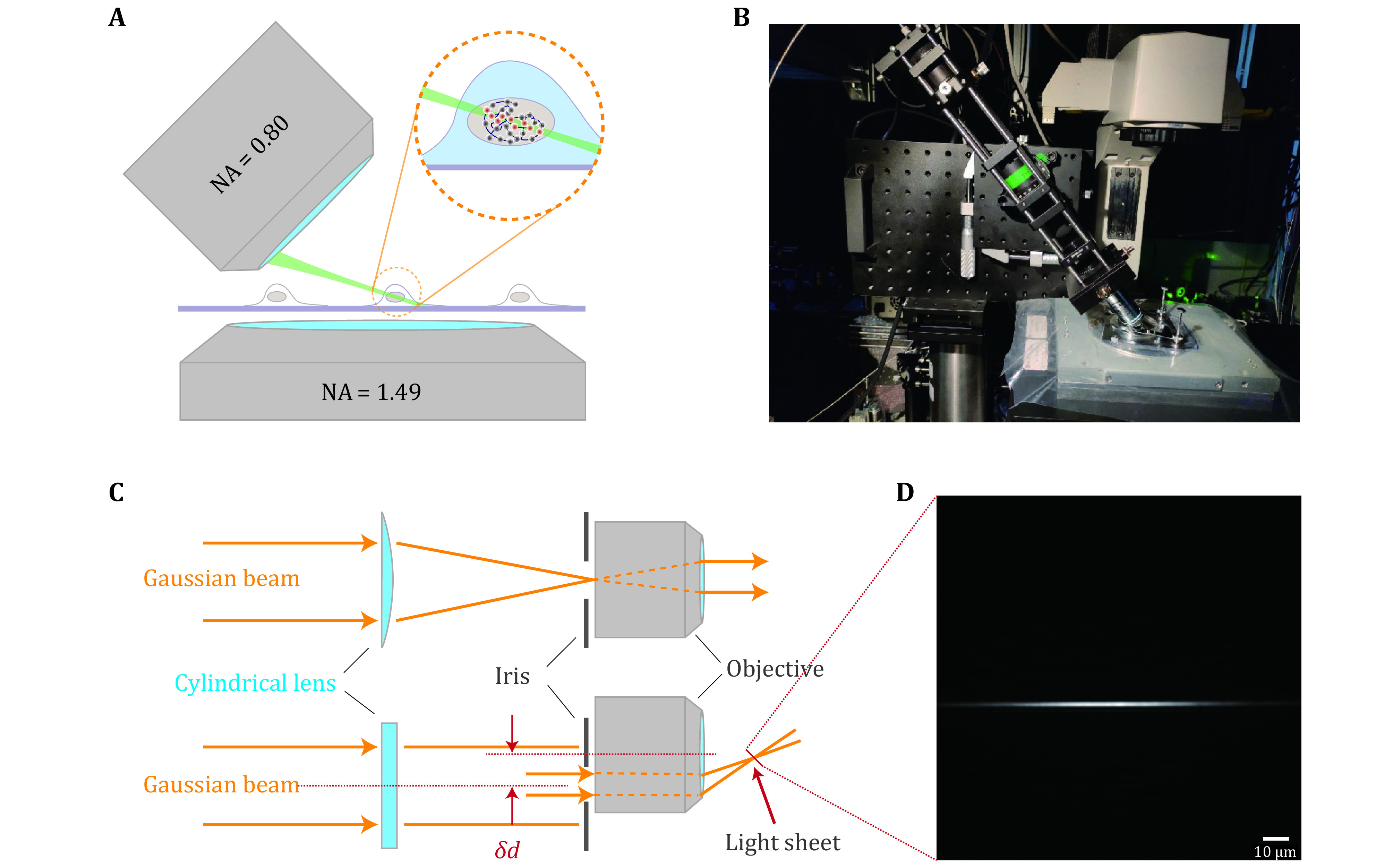
Principle and mechanical design of ImTLSM. **A** Schematic diagram showing the illumination mode of ImTLSM. The dashed circle emphasizes the excitation effect under ImTLSM illumination mode. The red dots indicate the fluorophores that were excited while the gray ones were not. **B** Photograph of the ImTLSM illumination part. **C** Schematic diagram of ImTLSM illumination principle. The Gaussian beam was formed through a laser combination system and a single-mode fiber, and was pre-collimated by a lens before directed into the illumination system. A cylindrical lens (focus length = 150 mm) was used to form a sheet pattern on the back-focal-plane of the illumination objective, where an adjustable iris was used for adjustment of the final FWHM of light sheet. **D** Light sheet profile along vertical direction. The image was taken by directing the light sheet imaged on the EMCCD (560 nm, 60× magnification in total)

We chose to form the light-sheet through a cylindrical lens. Distinct from common selective plane illumination microscopy (SPIM) (Huisken *et al*. 2004), the illumination objective (NIR Apo 40×/0.80 W DIC NI ∞/0 WD 3.5, Nikon) of ImTLSM was placed about 5 mm shift laterally from the optical axis (Fig. 6C). Analogous to highly inclined and laminated optical sheet (HILO) microscopy (Tokunaga *et al*. 2008), the incident *y*-axis focused Gaussian beam was positioned to the edge of the objective, resulting in the output of a tilted light-sheet from the objective. A graduated iris (SM1D12C, Thorlabs) was mounted at the back-focal-plane of the illumination objective to adjust the thickness of light sheet. The illumination system was rotated 45 degrees and mounted on a translation system to facilitate adjustment of the light-sheet position (Fig. 6B). An extra block was used to avoid ImTLSM illumination from reaching the EMCCD directly. Under ImTLSM illumination, the cell at the center of FOV was optically sectioned by the tilted light sheet (Fig. 6A) to remove the out-of-focus background physically. We note that this light-sheet system enables freely region-of-interest (ROI) searching like a common inverted microscope without further manipulations, which is crucial for high-throughput applications (supplementary Movie S1). In addition, such configuration allows a high NA objective (NA = 1.49) for sensitive single molecule detection (Fig. 6A). Therefore, ImTLSM is a robust light-sheet system which is particularly useful for high throughput intra-nucleus imaging. To use the full optical sectioning ability, we used the “iris-all-open” illumination mode under which the highest SBR could be achieved. On the other hand, we utilized illumination mode with iris diameter of 1.5 mm for trade-off between relatively high SBR and robustness of illumination for a range of *z*-axis positions. This mode helped to facilitate the gathering of datasets for deep-learning.

### Large-field homogeneous illumination

ImTLSM has a relatively small FOV (see Section of Characterization of ImTLSM performance). We therefore introduced large-field homogeneous illumination (Deschamps *et al*. 2016; Douglass *et al*. 2016; Stehr *et al*. 2019; Voelkel and Weible 2008; Zhao *et al*. 2017) to increase the effective FOV and throughput of ImTLSM-facilitated background free imaging. To do so, we coupled the free-space output of lasers into a customized multimode fiber, which is 20 meter long with a 200 μm^2^ square core, a 238 μm cladding diameter, and FC/APC connectors at both ends (WFANS 200 × 200 / 238 × 238, CeramOptec). The NA of the square-core fiber was 0.22. The optical fiber was bended into a circle with a radius of 15 cm, and bound to a vibration motor with high frequency (~11,000 r/min) to remove speckles. The optical system was built on a Nikon Eclipse Ti-E inverted microscope. Three lasers with wavelengths of 642 nm (2RU-VFL-P-1000-642-B1R, MPB Communications), 560 nm (2RU-VFL-P-1000-560-BIR, MPB Communications) and 488 nm (2RU-VFL-P-300-488-BIR, MPB Communications) were used to switch off fluorophores, while a 405 nm laser (OBIS, Coherent) was used to control the activation rate of the fluorophores to the fluorescence-emitting state. Each laser beam was reflected by an aluminum mirror and a dichroic mirror, then pointed into an AOTF (AODS20160, Crystal Technology). Finally, laser beams were focused into a fiber adapter (SM1FCA, Thorlabs) and coupled into the custom multimode fiber. The output beams passed through an objective lens (10×/NA 0.25, Olympus) then illuminated the sample by an objective lens (Plan Apo 100 × 1.49 Oil immersion, Nikon). Emitted fluorescence from the sample was collected by the same objective, filtered with a dichroic mirror (Di01-R405/488/561/635, Semrock) and an emission filter (FF01-446/523/600/677-25, Semrock), and was imaged by a tube lens (*f* = 200 mm, MXA20696, Nikon) onto an EMCCD camera (DU-897D, Andor). The width of a square camera pixel corresponds to 157 nm on the sample. Detailed imaging procedure could be found elsewhere (Betzig *et al*. 2006; Hess *et al*. 2006; Rust *et al*. 2006).

### Sample preparation

#### Cell culture and manipulations for H2B-mMaple3 stable cell line

HeLa S3 cells were maintained in Dulbecco’s modified Eagle medium with high glucose (Lifetech), 10% fetal bovine serum (FBS) (Lifetech), 1× penicillin/streptomycin (Lifetech) at 37 °C and 5% CO_2_ in dishes. Human H2B gene and fluorescent protein mMaple3 gene were cloned into the plasmid pcDNA 3.1(+) and 500 μg of the plasmids was transfected into HeLa cells with Lipofectamine 2000. After one-day growth, cells were constantly treated with G418 at the concentration of 200 μg/mL for two weeks. Positive clones with fluorescence were sorted and collected by flow cytometry (BD Biosciences).

#### Immunostaining

For immunostaining labeling of H2B, cells were first plated to dishes and grew for 24 h. Then the cells were fixed using 4% paraformaldehyde (PFA) in PBS for 15 min, followed by washing three times in 1× PBS at room temperature for 5 min each time. Cells were then permeabilized using 0.5% Triton X-100 (Sigma Aldrich, X100) in PBS for 10 min. Blocking was performed using 5% IgG-free Bovine Serum Albumin (BSA) for 30 min. Primary antibodies for H2B (Abcam; ab1790; 0.5 µg/mL) was added to 5% BSA in PBS and incubated overnight at 4 °C, washed with PBS three times (5 min). Cells were then incubated with secondary antibody (Invitrogen; A-21202; 1 µg/mL) at a concentration of 1∶200 in PBS with 5% BSA for 1 h. Finally, cells were washed with PBS for three times (5 min at room temperature for each time) and fixed using 4% PFA in PBS for 10 min. After rinsing twice with PBS, the cells were stored in PBS (4 °C).

For fluorescence labeling of cellular structures, the antibodies and concentrations were as following: for NCL, rabbit polyclonal anti-NCL (Abcam; ab22758, 1∶1000), Alexa Fluor 594 donkey anti-rabbit IgG (H+L) (Thermo Fisher Scientific; A21207; 1∶1000); for Lamin B1, Anti-Lamin B1 Mouse Monoclonal Antibody (Easybio; #BE3168-100; 1∶200), Alexa Fluor 594 goat anti-mouse IgG (H+L) (Thermo Fisher Scientific; A11032; 1∶1000); for Nup133, anti-Nup133 (Abcam; ab155990; 1∶200), Alexa Fluor 594 donkey anti-rabbit IgG (H+L) (Thermo Fisher Scientific; A21207; 1∶1000).

#### YAP-HaloTag labeling

Mouse embryonic fibroblast cell line MEF were maintained in Dulbecco’s modified Eagle medium (DMEM) with high glucose (Lifetech), 10% FBS (Lifetech), 1X penicillin/streptomycin (Lifetech). Cells were maintained at 37 °C and 5% CO_2_ in a humidified incubator. One day before transfection, cells were inoculated into 50 mm petri-dish (WillCo Wells, GWST-5040) with about 50% density. The next day, YAP-HaloTag plasmid was transiently transfected with Lipofectamine 2000 (Lifetech) in accordance with the manufacturer’s protocol. Then cells were grown overnight on 50 mm petri-dish overnight. Cells were labeled with Halo-JF549 dye at a final concentration of 0.1 nmol/L for 15 min and then washed three times with 1× PBS. After the final wash, PBS were replaced with fresh phenol red-free medium (Lifetech) for imaging.

### Data acquisition

#### Calibration of ImTLSM illumination profile

To calibrate the ImTLSM illumination profile, we projected the light-sheet of minimal power output directly to the EMCCD (Fig. 6D). To do so, we changed the shift direction of the illumination objective so that the light sheet was directed to the detection path of the microscope. The tilted angle was then the surplus angle for the normally used tilted angle. To avoid the reflection and interference induced by the coverslip, we put the focal plane of the light sheet several hundreds of micrometers above the coverslip. An objective (S PLAN Fluorescence ELWD 40×/0.60 OFN22 DIC NI) with long working distance was used. Dichroic mirror and emission filters were removed from the light path. We used 560 nm laser for light-sheet profile characterization as it is in the middle of illumination wavelength range. The light-sheet profile was imaged at 30 ms per frame with 60× magnification without EM gain. *Z*-axis scanning process was performed from bottom (10.0 μm below the light-sheet focus) to top (10.0 μm above the light-sheet focus), with a step size of 0.5 μm. 100 frames were taken and averaged for each *z*-position. We used Gaussian function to fit the profile in one direction (custom code in Python). The lateral position and FWHM were thus both calibrated along the *z*-axis of the detection objective (Fig. 1B).

#### Fluorescence imaging of cellular structures (NCL, Lamin B1, Nup133)

We imaged NCL structure to evaluate the performance of ImTLSM for different imaging demands. For the sectioning ability of ImTLSM, a water objective lens (Plan Apo 60×A/1.20 WI ∞/0.15-0.18 WD 0.31-0.28 OFN25 DIC N2 MRD07602) was used with a total 90× magnification to reduce the aberration caused by refraction index mismatch. 20 frames were taken for each *z*-position (30 ms per frame, 200 EM gain). To compare the SBR between light-sheet illumination mode and epi-illumination mode, an oil objective lens (Plan Apo 100 × 1.49 Oil immersion, Nikon) was used for high fluorescence collection efficiency (30 ms per frame for 100 frames with 100× magnification, 50 EM gain). The SBR of NCL was defined as the edge signal divided by the center part background intensity, which was calculated using custom Python code. For imaging of Lamin B1 and Nup133, we also chose the same oil objective lens (30 ms per frame with 100× magnification, 200 EM gain).

#### Single-molecule tracking

For single-molecule tracking of YAP-HaloTag, we chose an oil objective lens (Plan Apo 100 × 1.49 Oil immersion, Nikon) for high fluorescence collection efficiency (30 ms per frame with 100× magnification, 200 EM gain). The SBR was calculated using Insight3 and custom Python code.

#### Collection of deep learning datasets

For cross-modality training, we collected image pairs of the same FOV under ImTLSM illumination mode (iris diameter: 1.5 mm) and epi-illumination mode (50 ms per frame for 100 frames image, with 100× magnification, 50 EM gain). 1,000 FOVs were selected and collected. The entire data collection process, including search, focusing, and imaging of hundreds of cells, only took 20 hours, demonstrating the high throughput capability of ImTLSM. The image pairs were then pre-processed to form training dataset (see Section of Data preparation below for details).

#### Large-field homogeneous illumination imaging

For large-field homogeneous illumination imaging of H2B immuno-stained sample, we chose an oil objective lens (Plan Apo 100 × 1.49 Oil immersion, Nikon) for high fluorescence collection efficiency (50 ms per frame with 100× magnification, 200 EM gain).

#### SMLM imaging

For large FOV SMLM imaging of H2B-mMaple3 sample, we chose an oil objective lens (Plan Apo 100 × 1.49 Oil immersion, Nikon) for high fluorescence collection efficiency (30 ms per frame with 100× magnification, 100 EM gain). The power density of 560 nm laser was above 1 kW/cm^2^ over the 63 × 63 μm^2^ illumination area. A low intensity 405 nm laser was used to activate the probes before SMLM data acquisition, and was constantly adjusted during data acquisition to maintain a proper activation rate of fluorescent probes at any given time.

### Modified PReNet structure and training

We collected a series of image sequence pairs corresponding to epi-illumination mode and ImTLSM mode, respectively. We utilized a lightweight network named progressive recurrent network (PReNet) (Ren *et al*. 2019) for cross-modality training from epi-illumination images to ImTLSM images, thus to approach lightsheet-level background removal under conventional high-throughput epi-illumination imaging mode. In practice, pre-processed low noise conventional images were treated as input to the pre-trained network, then came with the final debackground output image.

#### Data preparation

The imaging regions at the illumination center of ImTLSM (about 5 μm in width) were first cropped from original images. 100 frames were averaged to reduce noise, which ensured that the model could learn the background without obstruction from the noise. 2,400 averaged image pairs were cropped from 800 high-quality image pairs, which were used as training dataset.

#### Model architecture

We developed debackground models by modifying an existing lightweight deraining neural network named as PReNet (Progressive Recurrent Network). PReNet was originally designed to tackle the training problems in multiple stage rather than building deeper networks. Regarding the modification on PReNet, we added Batch Normalization (BN) layer at the beginning of PReNet (M-PReNet) to prevent overfitting and accelerate deep network training (Ioffe *et al*. 2015). Similar operations have been reported in DeepSTORM3D (Nehme *et al*. 2020), in which BN layer was placed at the beginning of the architecture as a regularizer. In our work, adding BN layer at the beginning of PReNet helped our network learn the right normalization of the input images. We found that our modified PReNet (M-PReNet) performed the best among different networks that we tested (supplementary Fig. S8). The whole network could be separated to four parts, denoted as $ {f}_{in} $, $ {f}_{LSTM} $, $ {f}_{Res} $, $ {f}_{out} $, respectively.These four parts share the same parameters across different stages. The training processes were presented in Fig. 3. The input image was firstly passed through a BN-Conv-ReLU block, which was represented by the $ {f}_{in} $. The following $ {f}_{LSTM} $ means a convolutional Long Short-Term Memory (LSTM) unit (Shi *et al*. 2015) and $ {f}_{Res} $ contains five residual nets (ResNet) blocks stacked (He *et al*. 2016). Finally, $ {f}_{out} $ stand for a 1 × 1 Conv layer from which we obtained the output image.

#### Optimization

We chose mean-square error (MSE) as our loss function:

$ \mathcal{L}={||{\rm{Network}}\left(x\right)-{x}^{{\rm{gt}}}||}^{2} ,$

where “${\rm{Network}}$” represents the modified PReNet in Fig. 3, “$x^{{\rm{gt}}}$” represents the ground truth, “*x*” represents the input image. Parameters used in Adam optimizer were listed in Table 1. All computations were implemented by pytorch. The trainable parameters of the optimizer were initialized by the default settings in pytorch. Training was executed on NVidia 1080Ti GPU with 11 GB of video memory and evaluation was run on ThinkStation equipped with 128 GB of memory, Intel Xeon CPU E5-2620. The time consumption during our training and validating process were shown in supplementary Table S1.

**Table 1:** Parameters used in Adam optimizer

Name	Value
Lr	$ 1\times {10}^{-3} $
Beta1	0.9
Beta2	0.999
Epsilon	$ {10}^{-8} $
Batch size	128

### SMLM data analysis

Before single-molecule localization analysis, we first used PURE-LET (Li *et al*. 2018) to reduce the Poisson-Gaussian noise. Then background removal was performed either by applying pre-trained deep learning model or using ImageJ (Subtract background plugin, rolling ball radius of 50.0 pixels). SMLM analysis was performed using ThunderSTORM (Ovesný *et al*. 2014). We chose “local maximum” method with peak intensity threshold of 4*std (Wave.F1). Weighted Least squares fitting method was used with multi-emitter fitting analysis enabled. The photons were limited to intensity range of 500:2500 to reduce artifacts, especially for background removal by ImageJ plugin. Drift was corrected using the cross-correlation in ThunderSTORM with magnification of five. Duplicate localizations were removed in ThunderSTORM with distance threshold of 30 nm.

## Conflict of interest

Boxin Xue, Caiwei Zhou, Yizhi Qin, Yongzheng Li, Yuao Sun, Lei Chang, Shipeng Shao, Yongliang Li, Mengling Zhang, Chaoying Sun, Renxi He, Qian Peter Su and Yujie Sun declare that they have no conflict of interest.
